# Redetermination of [Pr(NO_3_)_3_(H_2_O)_4_]·2H_2_O

**DOI:** 10.1107/S1600536812028024

**Published:** 2012-06-27

**Authors:** Roel Decadt, Pascal Van Der Voort, Isabel Van Driessche, Rik Van Deun, Kristof Van Hecke

**Affiliations:** a Department of Inorganic and Physical Chemistry, Ghent University, Krijgslaan 281 – S3, B-9000 Ghent, Belgium

## Abstract

The structure of the title compound, tetra­aqua­tris­(nitrato-κ^2^ *O*,*O*′)praseodymium(III) dihydrate, was redetermined. The structure models derived from the previous determinations [Rumanova *et al.* (1964 ▶). *Kristallografiya*, **9**, 642–654; Fuller & Jacobson (1976 ▶). *Cryst. Struct. Commun.* **5**, 349–352] were confirmed, but now with all H atoms unambiguously located, revealing a complex O—H⋯O hydrogen-bonding network, extending throughout the whole structure. In the title compound, the coordination environment of the Pr^III^ atom can best be described as a distorted bicapped square anti­prism defined by three bidentate nitrate anions and four water mol­ecules. Additionally, two lattice water mol­ecules are observed in the crystal packing. The title compound is isotypic with several other lanthanide-containing nitrate analogues.

## Related literature

For general background and the synthesis of the title compound, see: Liu *et al.* (2012 ▶). For the original determined structures, see: Fuller & Jacobson (1976 ▶); Rumanova *et al.* (1964 ▶). For analogous *Ln*-containing structures (*Ln* = lanthanide), see: Kawashima *et al.* (2000 ▶); Rogers *et al.* (1983 ▶); Shi & Wang (1990 ▶, 1991 ▶); Stumpf & Bolte (2001 ▶). For related structures of metal-organic compounds, see: Rohde & Urland (2006 ▶); Weakley (1982 ▶, 1984 ▶, 1989 ▶). For databases of (in)organic structures, see: Allen (2002 ▶); Bergerhoff *et al.* (1983 ▶); ICSD (2009 ▶).

## Experimental

### 

#### Crystal data

[Pr(NO_3_)_3_(H_2_O)_4_]·2H_2_O
*M* *_r_* = 435.04
Triclinic, 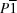

*a* = 6.7017 (3) Å
*b* = 9.1858 (4) Å
*c* = 11.7010 (6) Å
α = 69.118 (4)°
β = 88.958 (4)°
γ = 69.696 (4)°
*V* = 626.60 (6) Å^3^
*Z* = 2
Mo *K*α radiation
μ = 3.98 mm^−1^
*T* = 100 K
0.37 × 0.17 × 0.14 mm

#### Data collection

Agilent SuperNova diffractometer with an Atlas detector
Absorption correction: numerical [*CrysAlis PRO* (Agilent, 2010 ▶), using a multi-faceted crystal model based on expressions derived by Clark & Reid (1995 ▶)] *T*_min_ = 0.375, *T*_max_ = 0.654
10260 measured reflections
2743 independent reflections
2627 reflections with *I* > 2σ(*I*)
*R*_int_ = 0.035

#### Refinement

*R*[*F* ^2^ > 2σ(*F* ^2^)] = 0.017
*wR*(*F* ^2^) = 0.037
*S* = 1.07
2743 reflections
208 parameters
12 restraints
All H-atom parameters refined
Δρ_max_ = 0.42 e Å^−3^
Δρ_min_ = −0.60 e Å^−3^

### 

Data collection: *CrysAlis PRO* (Agilent, 2010 ▶); cell refinement: *CrysAlis PRO*; data reduction: *CrysAlis PRO*; program(s) used to solve structure: *SHELXS97* (Sheldrick, 2008 ▶); program(s) used to refine structure: *SHELXL97* (Sheldrick, 2008 ▶); molecular graphics: *DIAMOND* (Brandenburg, 2008 ▶); software used to prepare material for publication: *PLATON* (Spek, 2009 ▶).

## Supplementary Material

Crystal structure: contains datablock(s) I, global. DOI: 10.1107/S1600536812028024/wm2647sup1.cif

Structure factors: contains datablock(s) I. DOI: 10.1107/S1600536812028024/wm2647Isup2.hkl

Additional supplementary materials:  crystallographic information; 3D view; checkCIF report

## Figures and Tables

**Table 1:** Selected bond lengths (Å)

O1—Pr1	2.5677 (16)
O2—Pr1	2.5790 (15)
O4—Pr1	2.6348 (16)
O5—Pr1	2.6000 (17)
O7—Pr1	2.7307 (15)
O8—Pr1	2.6154 (16)
O10—Pr1	2.4468 (17)
O11—Pr1	2.4287 (17)
O12—Pr1	2.4555 (16)
O13—Pr1	2.4578 (17)

**Table 2:** Hydrogen-bond geometry (Å, °)

*D*—H⋯*A*	*D*—H	H⋯*A*	*D*⋯*A*	*D*—H⋯*A*
O10—H10*A*⋯O8^i^	0.82 (2)	2.10 (2)	2.918 (2)	177 (3)
O10—H10*B*⋯O14^ii^	0.82 (2)	1.85 (2)	2.670 (2)	176 (3)
O11—H11*A*⋯O14^i^	0.80 (2)	1.94 (2)	2.735 (2)	177 (3)
O11—H11*B*⋯O15^iii^	0.80 (2)	1.93 (2)	2.720 (2)	175 (3)
O12—H12*A*⋯O4^iv^	0.81 (2)	2.11 (2)	2.925 (2)	174 (3)
O12—H12*B*⋯O15	0.81 (2)	1.91 (2)	2.713 (2)	175 (3)
O13—H13*A*⋯O4^ii^	0.81 (2)	2.38 (2)	3.137 (2)	156 (3)
O13—H13*A*⋯O8^ii^	0.81 (2)	2.57 (3)	3.130 (2)	128 (3)
O13—H13*B*⋯O7^v^	0.79 (2)	2.24 (2)	3.020 (2)	168 (3)
O13—H13*B*⋯O9^v^	0.79 (2)	2.43 (2)	3.046 (2)	136 (3)
O14—H14*A*⋯O9	0.84 (2)	2.00 (2)	2.826 (2)	169 (3)
O14—H14*B*⋯O5^vi^	0.80 (2)	2.26 (2)	2.881 (2)	134 (3)
O14—H14*B*⋯O3^vii^	0.80 (2)	2.39 (2)	2.971 (2)	130 (3)
O15—H15*B*⋯O6^ii^	0.83 (2)	2.01 (2)	2.824 (2)	169 (3)
O15—H15*A*⋯O3^iv^	0.77 (2)	2.56 (2)	3.049 (2)	123 (2)
O15—H15*A*⋯O3^viii^	0.77 (2)	2.28 (2)	2.892 (2)	138 (3)

Symmetry codes: (i) 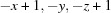; (ii) 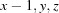; (iii) 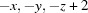; (iv) 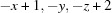; (v) 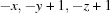; (vi) 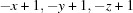; (vii) 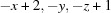; (viii) 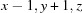.

**Fig. 1.**
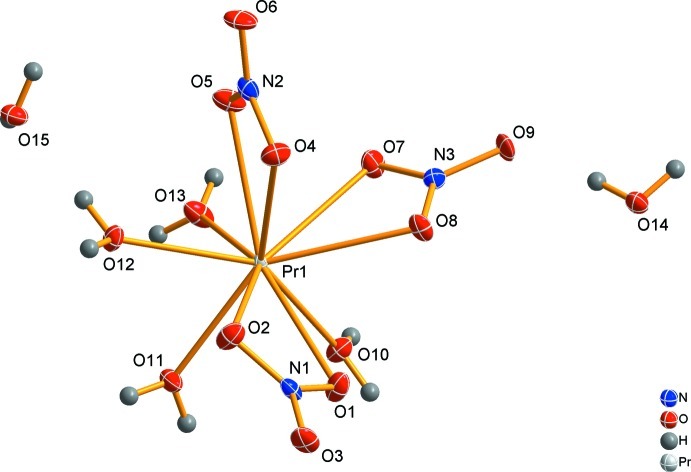
Coordination geometry of the title compound, showing the atom-labelling scheme of the asymmetric unit and 60% probability displacement ellipsoids.

**Fig. 2.**
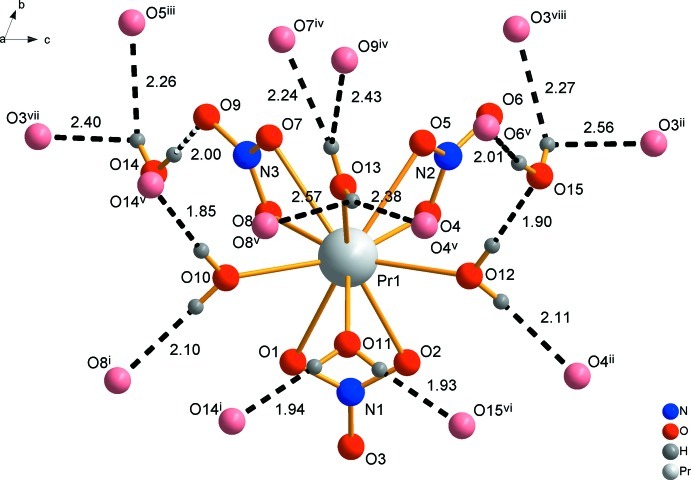
Extended hydrogen bond network in the structure of the title compound, with atom-labeling scheme. Hydrogen bonds are indicated. Symmetry equivalent oxygen atoms are colored pink. Symmetry codes (i) 1 - x,-y,1 - z; (ii) 1 - x,-y,2 - z; (iii) 1 - x,1 - y,1 - z; (iv) -x,1 - y,1 - z; (v) -1 + x,y,z; (vi) -x,-y,2 - z; (vii) 2 - x,-y,1 - z; (viii) -1 + x,1 + y,z.

**Fig. 3.**
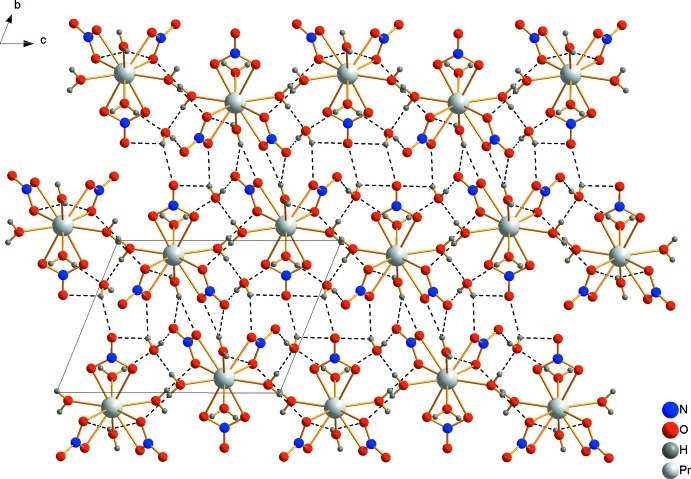
Packing diagram of the title compound along the crystallographic a-axis, indicating the extended hydrogen bond network.

## supplementary crystallographic information

### Comment

The title compound was serendipitously obtained in low yield as an undesired product of an experiment aimed at obtaining a Pr^III^-containing coordination polymer, with dicarboxylate ligands as the connecting moieties, following an earlier successful synthesis of a vanadium metal-organic framework with the same type of linkers (Liu *et al.*, 2012).

The structure of the title compound is analogous to the structures, previously determined, with ICSD entries 22339 (Rumanova *et al.*, 1964) and 123 (Fuller & Jacobson, 1976) (ICSD Version 1.8.1; Bergerhoff *et al.*, 1983; ICSD, 2009). However, in both of the latter structures, the "position of elements of H were undetermined". In the now determined structure, these hydrogen atoms could unambiguously be located from difference Fourier electron density maps, revealing an extended hydrogen bonding network.

The structure of the title compound is isotypic with other [*Ln*(NO_3_)_3_(H_2_O)_4_]^.^2H_2_O analogues, for example for *Ln* = Nd (ICSD entries 37181 and 71767) (Rogers *et al.*, 1983; Shi & Wang, 1991), *Ln* = Sm (ICSD entry 69158) (Shi & Wang, 1990) and *Ln* = Eu (ICSD entry 280528) (Stumpf & Bolte, 2001). Three additional Sm-analogues were reported by Kawashima *et al.*, 2000 (ICSD entries 91511, 91512 and 91513).

The asymmetric unit consists of one Pr^III^ cation, three nitrate anions, and in total six water molecules. The Pr^III^ cation is ten-coordinated by the oxygen atoms of three bidentate nitrate anions and four water molecules. Additionally, two lattice water molecules are included in the second coordination sphere of the praseodymium cation. The coordination polyhedron around Pr^III^ can be best described as a distorted bicapped square antiprism (Figure 1). The Pr—O distances range from 2.5677 (16) to 2.7307 (15) Å and from 2.4287 (17) to 2.4578 (17) Å for the coordinating nitrate groups and water molecules, respectively (Table 1). The coordinating nitrate groups are positioned on the same side of the polyhedron, whereas the coordinating water molecules are positioned on the opposite side.

When searching the Cambridge Structural Database (CSD, Version 5.33) (Allen, 2002), another Pr^III^-complex is found (CSD reference code VAFLOD), containing three nitrate anions and four water molecules in its coordination sphere. However, this complex shows a totally different, approximately twofold symmetry (Weakley, 1989). Other structures of metal-organic complexes of Pr^III^, coordinated by only nitrate and water molecules are found, showing different coordination assemblies, *i.e.* a 12-coordinated Pr^III^ atom with five nitrate and two aqua ligands, balanced by two additional counter ions (CSD reference code QERRIP) (Rohde & Urland, 2006), a complex with three nitrate and three aqua ligands (CSD reference code CUKMUQ) (Weakley, 1984), and even the formation of dimers (CSD reference code BUPFIB) (Weakley, 1982) have been previously reported.

In the reported structure, a complex, extended hydrogen bonding network is formed throughout the whole structure, stabilizing the crystal packing, *i.e.* in total 16 different hydrogen bonds are observed between the coordinating water molecules, nitrate anions and lattice water molecules (Figure 2). In total, eight different symmetry equivalent water or nitrate oxygen atoms are involved in the hydrogen bond network. In fact, the praseodymium complexes are all interconnected through these solvent water molecule hydrogen bonds (Figure 3). The four coordinating water molecules show the following hydrogen bonds: O10 is hydrogen bonded to the symmetry-equivalent water molecule oxygen atom O14 and nitrate O8. Water O11 is hydrogen bonded to the symmetry equivalent waters O14 and O15. Water O12 is hydrogen bonded to the symmetry equivalent nitrate O4 and forms an intramolecular hydrogen bond with water O15. Water O13 shows two bifurcated hydrogen bonds to the symmetry equivalent nitrates O7 and O9 and to O4 and O8. The solvent water molecule O14 forms an intramolecular hydrogen bond with nitrate O9 and a bifurcated hydrogen bond to the symmetry equivalent nitrates O3 and O5. Solvent water molecule O15 forms a bifurcated hydrogen bond to two different symmetry equivalent nitrate O3 atoms and another hydrogen bond to a symmetry equivalent nitrate O6. Details of the hydrogen-bonding geometry are given in Table 2.

### Experimental

The title compound was serendipitously obtained in low yield as a product of an experiment aimed at obtaining a Pr^III^-containing coordination polymer, with dicarboxylate ligands as the connecting moiety, following an earlier successful synthesis of a vanadium metal-organic framework with the same type of linkers (Liu *et al.*, 2012).

In the synthesis, 0.1 mmol Pr(NO_3_)_3_^.^xH_2_O, along with 0.3 mmol dicarboxylic acid and four drops of 0.6 *M* aqueous HNO_3_ was dissolved in 5 ml of a 1:1 mixture of 1:1 MeOH/H_2_O. After seven days of heating the mixture to 363 K, the title compound was isolated as colourless crystals, suitable for single-crystal X-ray diffraction analysis.

### Refinement

All hydrogen atoms were located in a difference Fourier electron density map and further refined with isotropic temperature factors fixed at 1.5 times *U*_eq_ of the parent atoms, applying a restraint value of 0.84 (2) Å for the O—H distances.

### Crystal data

**Table:**

[Pr(NO_3_)_3_(H_2_O)_4_]·2H_2_O	*Z* = 2
*M**_r_* = 435.04	*F*(000) = 424
Triclinic, *P*1	*D*_x_ = 2.306 Mg m^−^^3^
Hall symbol: -P 1	Mo *K*α radiation, λ = 0.71073 Å
*a* = 6.7017 (3) Å	Cell parameters from 8040 reflections
*b* = 9.1858 (4) Å	θ = 3.3–29.3°
*c* = 11.7010 (6) Å	µ = 3.98 mm^−^^1^
α = 69.118 (4)°	*T* = 100 K
β = 88.958 (4)°	Rod, colourless
γ = 69.696 (4)°	0.37 × 0.17 × 0.14 mm
*V* = 626.60 (6) Å^3^	

### Data collection

**Table:**

Agilent SuperNova diffractometer with an Atlas detector	2743 independent reflections
Radiation source: SuperNova (Mo) X-ray Source	2627 reflections with *I* > 2σ(*I*)
Mirror monochromator	*R*_int_ = 0.035
Detector resolution: 10.3693 pixels mm^-1^	θ_max_ = 27.1°, θ_min_ = 3.3°
ω scans	*h* = −8→8
Absorption correction: numerical [*CrysAlis PRO* (Agilent, 2010), using a multi-faceted crystal model based on expressions derived by Clark & Reid (1995)]	*k* = −11→11
*T*_min_ = 0.375, *T*_max_ = 0.654	*l* = −14→14
10260 measured reflections	

### Refinement

**Table:**

Refinement on *F*^2^	Primary atom site location: structure-invariant direct methods
Least-squares matrix: full	Secondary atom site location: difference Fourier map
*R*[*F*^2^ > 2σ(*F*^2^)] = 0.017	Hydrogen site location: inferred from neighbouring sites
*wR*(*F*^2^) = 0.037	All H-atom parameters refined
*S* = 1.07	*w* = 1/[σ^2^(*F*_o_^2^) + (0.0119*P*)^2^ + 0.0612*P*] where *P* = (*F*_o_^2^ + 2*F*_c_^2^)/3
2743 reflections	(Δ/σ)_max_ = 0.001
208 parameters	Δρ_max_ = 0.42 e Å^−^^3^
12 restraints	Δρ_min_ = −0.60 e Å^−^^3^

### Special details

**Table:**

Geometry. All e.s.d.'s (except the e.s.d. in the dihedral angle between two l.s. planes) are estimated using the full covariance matrix. The cell e.s.d.'s are taken into account individually in the estimation of e.s.d.'s in distances, angles and torsion angles; correlations between e.s.d.'s in cell parameters are only used when they are defined by crystal symmetry. An approximate (isotropic) treatment of cell e.s.d.'s is used for estimating e.s.d.'s involving l.s. planes.
Refinement. Refinement of *F*^2^ against ALL reflections. The weighted *R*-factor *wR* and goodness of fit *S* are based on *F*^2^, conventional *R*-factors *R* are based on *F*, with *F* set to zero for negative *F*^2^. The threshold expression of *F*^2^ > σ(*F*^2^) is used only for calculating *R*-factors(gt) *etc*. and is not relevant to the choice of reflections for refinement. *R*-factors based on *F*^2^ are statistically about twice as large as those based on *F*, and *R*- factors based on ALL data will be even larger.

### Fractional atomic coordinates and isotropic or equivalent isotropic displacement parameters (Å^2^)

**Table:**

	*x*	*y*	*z*	*U*_iso_*/*U*_eq_	
N1	0.6816 (3)	−0.2289 (2)	0.81616 (17)	0.0107 (4)	
N2	0.4207 (3)	0.3279 (2)	0.82332 (17)	0.0111 (4)	
N3	0.4305 (3)	0.3335 (2)	0.49700 (17)	0.0111 (4)	
O1	0.6030 (3)	−0.15602 (19)	0.70360 (14)	0.0153 (4)	
O2	0.5933 (3)	−0.15874 (19)	0.88855 (14)	0.0154 (4)	
O3	0.8379 (3)	−0.36072 (19)	0.85198 (15)	0.0159 (4)	
O4	0.5626 (3)	0.18920 (19)	0.82739 (15)	0.0146 (4)	
O5	0.2358 (3)	0.36386 (19)	0.77331 (14)	0.0147 (4)	
O6	0.4629 (3)	0.41926 (19)	0.86512 (15)	0.0168 (4)	
O7	0.2409 (3)	0.37864 (19)	0.52158 (15)	0.0155 (4)	
O8	0.5621 (2)	0.19139 (18)	0.56929 (14)	0.0124 (3)	
O9	0.4894 (3)	0.42152 (19)	0.40734 (14)	0.0156 (4)	
O10	0.2205 (3)	0.0527 (2)	0.54042 (15)	0.0124 (3)	
H10A	0.285 (4)	−0.016 (3)	0.510 (2)	0.019*	
H10B	0.128 (4)	0.119 (3)	0.483 (2)	0.019*	
O11	0.1239 (3)	−0.1023 (2)	0.77928 (15)	0.0149 (4)	
H11A	0.110 (5)	−0.154 (3)	0.740 (2)	0.022*	
H11B	0.122 (5)	−0.162 (3)	0.8474 (18)	0.022*	
O12	0.2015 (3)	0.0533 (2)	0.93368 (15)	0.0119 (3)	
H12A	0.268 (4)	−0.009 (3)	1.0013 (18)	0.018*	
H12B	0.112 (4)	0.126 (3)	0.951 (2)	0.018*	
O13	−0.0770 (3)	0.2713 (2)	0.67182 (16)	0.0159 (4)	
H13A	−0.175 (4)	0.241 (3)	0.695 (3)	0.024*	
H13B	−0.134 (4)	0.366 (2)	0.627 (2)	0.024*	
O14	0.9130 (3)	0.27933 (19)	0.35872 (15)	0.0121 (3)	
H14A	0.794 (3)	0.326 (3)	0.379 (2)	0.018*	
H14B	0.940 (4)	0.356 (3)	0.311 (2)	0.018*	
O15	−0.1154 (3)	0.2943 (2)	0.98338 (15)	0.0137 (4)	
H15B	−0.231 (3)	0.328 (3)	0.941 (2)	0.020*	
H15A	−0.075 (4)	0.366 (3)	0.976 (3)	0.020*	
Pr1	0.305901 (18)	0.096024 (13)	0.725397 (10)	0.00646 (5)	

### Atomic displacement parameters (Å^2^)

**Table:**

	*U*^11^	*U*^22^	*U*^33^	*U*^12^	*U*^13^	*U*^23^
N1	0.0087 (10)	0.0073 (9)	0.0132 (10)	−0.0035 (8)	0.0018 (8)	−0.0001 (8)
N2	0.0138 (10)	0.0104 (9)	0.0071 (9)	−0.0056 (8)	0.0001 (8)	−0.0001 (8)
N3	0.0115 (10)	0.0089 (9)	0.0126 (10)	−0.0032 (8)	0.0030 (8)	−0.0041 (8)
O1	0.0165 (9)	0.0132 (8)	0.0087 (8)	0.0011 (7)	0.0011 (7)	−0.0018 (7)
O2	0.0166 (9)	0.0125 (8)	0.0111 (8)	0.0016 (7)	−0.0011 (7)	−0.0043 (7)
O3	0.0125 (9)	0.0070 (7)	0.0201 (9)	0.0008 (7)	0.0021 (7)	−0.0002 (7)
O4	0.0136 (9)	0.0124 (8)	0.0174 (8)	−0.0023 (7)	0.0001 (7)	−0.0073 (7)
O5	0.0123 (8)	0.0088 (7)	0.0192 (9)	−0.0026 (6)	−0.0046 (7)	−0.0019 (7)
O6	0.0242 (10)	0.0121 (8)	0.0175 (9)	−0.0095 (7)	−0.0022 (7)	−0.0064 (7)
O7	0.0094 (9)	0.0123 (8)	0.0196 (9)	−0.0011 (7)	0.0050 (7)	−0.0031 (7)
O8	0.0091 (8)	0.0092 (7)	0.0117 (8)	−0.0011 (6)	−0.0009 (6)	0.0021 (6)
O9	0.0208 (9)	0.0103 (8)	0.0113 (8)	−0.0060 (7)	0.0076 (7)	0.0008 (7)
O10	0.0135 (9)	0.0107 (8)	0.0085 (8)	0.0007 (7)	−0.0019 (6)	−0.0033 (7)
O11	0.0270 (10)	0.0165 (9)	0.0085 (8)	−0.0152 (8)	0.0050 (7)	−0.0058 (7)
O12	0.0131 (9)	0.0118 (8)	0.0075 (8)	−0.0013 (7)	0.0015 (6)	−0.0028 (7)
O13	0.0081 (9)	0.0101 (8)	0.0238 (9)	−0.0025 (7)	−0.0007 (7)	−0.0005 (7)
O14	0.0136 (9)	0.0083 (8)	0.0122 (8)	−0.0036 (7)	0.0026 (7)	−0.0019 (7)
O15	0.0167 (9)	0.0107 (8)	0.0153 (9)	−0.0071 (7)	0.0013 (7)	−0.0048 (7)
Pr1	0.00633 (7)	0.00570 (7)	0.00600 (7)	−0.00178 (5)	0.00073 (5)	−0.00104 (5)

### Geometric parameters (Å, º)

**Table:**

N1—O3	1.230 (2)	O10—Pr1	2.4468 (17)
N1—O2	1.261 (3)	O10—H10A	0.823 (17)
N1—O1	1.270 (2)	O10—H10B	0.818 (17)
N1—Pr1	2.9996 (18)	O11—Pr1	2.4287 (17)
N2—O6	1.219 (2)	O11—H11A	0.795 (17)
N2—O5	1.261 (2)	O11—H11B	0.796 (17)
N2—O4	1.284 (2)	O12—Pr1	2.4555 (16)
N3—O9	1.229 (2)	O12—H12A	0.814 (16)
N3—O7	1.255 (2)	O12—H12B	0.810 (17)
N3—O8	1.280 (2)	O13—Pr1	2.4578 (17)
O1—Pr1	2.5677 (16)	O13—H13A	0.806 (17)
O2—Pr1	2.5790 (15)	O13—H13B	0.791 (17)
O4—Pr1	2.6348 (16)	O14—H14A	0.841 (17)
O5—Pr1	2.6000 (17)	O14—H14B	0.803 (17)
O7—Pr1	2.7307 (15)	O15—H15B	0.827 (17)
O8—Pr1	2.6154 (16)	O15—H15A	0.772 (17)
			
O3—N1—O2	121.92 (18)	O12—Pr1—O2	68.79 (5)
O3—N1—O1	121.3 (2)	O13—Pr1—O2	145.75 (6)
O2—N1—O1	116.81 (17)	O1—Pr1—O2	49.52 (5)
O3—N1—Pr1	178.82 (15)	O11—Pr1—O5	130.84 (6)
O2—N1—Pr1	58.64 (10)	O10—Pr1—O5	132.67 (5)
O1—N1—Pr1	58.17 (10)	O12—Pr1—O5	69.58 (5)
O6—N2—O5	122.21 (19)	O13—Pr1—O5	70.69 (5)
O6—N2—O4	121.75 (19)	O1—Pr1—O5	143.42 (5)
O5—N2—O4	116.04 (19)	O2—Pr1—O5	110.57 (5)
O9—N3—O7	121.79 (18)	O11—Pr1—O8	139.83 (6)
O9—N3—O8	120.80 (19)	O10—Pr1—O8	74.17 (5)
O7—N3—O8	117.42 (18)	O12—Pr1—O8	146.05 (5)
N1—O1—Pr1	96.98 (12)	O13—Pr1—O8	116.30 (5)
N1—O2—Pr1	96.69 (11)	O1—Pr1—O8	68.73 (5)
N2—O4—Pr1	96.44 (12)	O2—Pr1—O8	97.87 (5)
N2—O5—Pr1	98.76 (13)	O5—Pr1—O8	87.86 (5)
N3—O7—Pr1	94.96 (11)	O11—Pr1—O4	140.59 (5)
N3—O8—Pr1	99.87 (12)	O10—Pr1—O4	144.15 (5)
Pr1—O10—H10A	132.6 (19)	O12—Pr1—O4	76.01 (5)
Pr1—O10—H10B	127 (2)	O13—Pr1—O4	119.27 (6)
H10A—O10—H10B	99 (3)	O1—Pr1—O4	95.75 (5)
Pr1—O11—H11A	127 (2)	O2—Pr1—O4	68.85 (5)
Pr1—O11—H11B	125 (2)	O5—Pr1—O4	48.70 (5)
H11A—O11—H11B	101 (3)	O8—Pr1—O4	70.04 (5)
Pr1—O12—H12A	131 (2)	O11—Pr1—O7	130.94 (5)
Pr1—O12—H12B	123.3 (19)	O10—Pr1—O7	69.86 (5)
H12A—O12—H12B	101 (3)	O12—Pr1—O7	132.13 (5)
Pr1—O13—H13A	126 (2)	O13—Pr1—O7	69.00 (5)
Pr1—O13—H13B	130 (2)	O1—Pr1—O7	110.69 (5)
H13A—O13—H13B	104 (3)	O2—Pr1—O7	144.33 (5)
H14A—O14—H14B	104 (3)	O5—Pr1—O7	65.87 (5)
H15B—O15—H15A	112 (3)	O8—Pr1—O7	47.74 (5)
O11—Pr1—O10	70.89 (6)	O4—Pr1—O7	87.29 (5)
O11—Pr1—O12	70.61 (6)	O11—Pr1—N1	79.29 (6)
O10—Pr1—O12	139.73 (6)	O10—Pr1—N1	92.07 (5)
O11—Pr1—O13	75.51 (6)	O12—Pr1—N1	92.05 (5)
O10—Pr1—O13	78.75 (6)	O13—Pr1—N1	154.78 (5)
O12—Pr1—O13	80.71 (6)	O1—Pr1—N1	24.84 (5)
O11—Pr1—O1	80.55 (6)	O2—Pr1—N1	24.67 (5)
O10—Pr1—O1	68.87 (5)	O5—Pr1—N1	129.33 (5)
O12—Pr1—O1	115.35 (5)	O8—Pr1—N1	82.83 (5)
O13—Pr1—O1	144.58 (6)	O4—Pr1—N1	81.62 (5)
O11—Pr1—O2	79.84 (6)	O7—Pr1—N1	129.92 (5)
O10—Pr1—O2	114.99 (5)		

### Hydrogen-bond geometry (Å, º)

**Table:**

*D*—H···*A*	*D*—H	H···*A*	*D*···*A*	*D*—H···*A*
O10—H10*A*···O8^i^	0.82 (2)	2.10 (2)	2.918 (2)	177 (3)
O10—H10*B*···O14^ii^	0.82 (2)	1.85 (2)	2.670 (2)	176 (3)
O11—H11*A*···O14^i^	0.80 (2)	1.94 (2)	2.735 (2)	177 (3)
O11—H11*B*···O15^iii^	0.80 (2)	1.93 (2)	2.720 (2)	175 (3)
O12—H12*A*···O4^iv^	0.81 (2)	2.11 (2)	2.925 (2)	174 (3)
O12—H12*B*···O15	0.81 (2)	1.91 (2)	2.713 (2)	175 (3)
O13—H13*A*···O4^ii^	0.81 (2)	2.38 (2)	3.137 (2)	156 (3)
O13—H13*A*···O8^ii^	0.81 (2)	2.57 (3)	3.130 (2)	128 (3)
O13—H13*B*···O7^v^	0.79 (2)	2.24 (2)	3.020 (2)	168 (3)
O13—H13*B*···O9^v^	0.79 (2)	2.43 (2)	3.046 (2)	136 (3)
O14—H14*A*···O9	0.84 (2)	2.00 (2)	2.826 (2)	169 (3)
O14—H14*B*···O5^vi^	0.80 (2)	2.26 (2)	2.881 (2)	134 (3)
O14—H14*B*···O3^vii^	0.80 (2)	2.39 (2)	2.971 (2)	130 (3)
O15—H15*B*···O6^ii^	0.83 (2)	2.01 (2)	2.824 (2)	169 (3)
O15—H15*A*···O3^iv^	0.77 (2)	2.56 (2)	3.049 (2)	123 (2)
O15—H15*A*···O3^viii^	0.77 (2)	2.28 (2)	2.892 (2)	138 (3)

Symmetry codes: (i) −*x*+1, −*y*, −*z*+1; (ii) *x*−1, *y*, *z*; (iii) −*x*, −*y*, −*z*+2; (iv) −*x*+1, −*y*, −*z*+2; (v) −*x*, −*y*+1, −*z*+1; (vi) −*x*+1, −*y*+1, −*z*+1; (vii) −*x*+2, −*y*, −*z*+1; (viii) *x*−1, *y*+1, *z*.

## References

[bb1] Agilent (2010). *CrysAlis PRO* Agilent Technologies UK Ltd, Yarnton, England.

[bb2] Allen, F. H. (2002). *Acta Cryst.* B**58**, 380–388. 10.1107/s0108768102003890 12037359

[bb3] Bergerhoff, G., Hundt, R., Sievers, R. & Brown, I. D. (1983). *J. Chem. Inf. Comput. Sci.* **23**, 66–69.

[bb4] Brandenburg, K. (2008). *DIAMOND* Crystal Impact GbR, Bonn, Germany.

[bb5] Clark, R. C. & Reid, J. S. (1995). *Acta Cryst.* A**51**, 887–897.

[bb6] Fuller, C. & Jacobson, R. A. (1976). *Cryst. Struct. Commun.* **5**, 349–352.

[bb7] ICSD (2009). http://www.fiz-karlsruhe.de/icsd.html.

[bb8] Kawashima, R., Sasaki, M., Satoh, S., Isoda, H., Kino, Y. & Shiozaki, Y. (2000). *J. Phys. Soc. Jpn*, **69**, 3297–3303.

[bb9] Liu, Y. Y., Leus, K., Grzywa, M., Weinberger, D., Strubbe, K., Vrielinck, H., Van Deun, R., Volkmer, D., Van Speybroeck, V. & Van Der Voort, P. (2012). *Eur. J. Inorg. Chem.* pp. 2819–2827.

[bb10] Rogers, D. J., Taylor, N. J. & Toogood, G. E. (1983). *Acta Cryst.* C**39**, 939–941.

[bb11] Rohde, A. & Urland, W. (2006). *Acta Cryst.* E**62**, m3026–m3028.

[bb12] Rumanova, I. M., Volodina, G. F. & Belov, N. V. (1964). *Kristallografiya*, **9**, 642–654.

[bb13] Sheldrick, G. M. (2008). *Acta Cryst.* A**64**, 112–122. 10.1107/S0108767307043930 18156677

[bb14] Shi, B. D. & Wang, J. Z. (1990). *Jiegon Huaxue*, **9**, 164–167.

[bb15] Shi, B. D. & Wang, J. Z. (1991). *Xiamen Daxue Xuebao, Ziran Kexueban*, **30**, 55–58.

[bb16] Spek, A. L. (2009). *Acta Cryst.* D**65**, 148–155. 10.1107/S090744490804362X PMC2631630 19171970

[bb17] Stumpf, T. & Bolte, M. (2001). *Acta Cryst.* E**57**, i10–i11.

[bb18] Weakley, T. J. R. (1982). *Inorg. Chim. Acta*, **63**, 161–168.

[bb19] Weakley, T. J. R. (1984). *Inorg. Chim. Acta*, **95**, 317–322.

[bb20] Weakley, T. J. R. (1989). *Acta Cryst.* C**45**, 525–526.

